# Dexmedetomidine ameliorates muscle wasting and attenuates the alteration of hypothalamic neuropeptides and inflammation in endotoxemic rats

**DOI:** 10.1371/journal.pone.0174894

**Published:** 2017-03-30

**Authors:** Minhua Cheng, Tao Gao, Fengchan Xi, Chun Cao, Yan Chen, Chenyan Zhao, Qiurong Li, Wenkui Yu

**Affiliations:** 1 Medical School of Nanjing University,Nanjing, Jiangsu, P.R China; 2 Institute of General Surgery Jinling Hospital, Medical School of Nanjing University, Nanjing, Jiangsu, P.R China; The University of Tokyo, JAPAN

## Abstract

Dexmedetomidine is generally used for sedaton in critically ill, it could shorten duration of mechanical ventilation, ICU stay and lower basic metabolism. However, the exact mechanism of these positive effects remains unkown. Here we investigated the hypothesis that dexmedetomidine could ameliorate muscle wasting in endotoxemic rats and whether it was related to hypothalamic neuropeptides alteration and inflammation. Fourty-eight adult male Sprague–Dawley rats were intraperitoneally injected with lipopolysaccharide (LPS) (5 mg/kg) or saline, followed by 50 μg/kg dexmedetomidine or saline administration via the femoral vein catheter (infusion at 5 μg·kg^-1^·hr^-1^). Twenty-four hours after injection, hypothalamus tissues and skeletal muscle were obtained. Muscle wasting was measured by the mRNA expression of two E3 ubiquitin ligases, muscle atrophy F-box (MAFbx) and muscle ring finger 1 (MuRF-1) as well as 3-methylhistidine (3-MH) and tyrosine release. Hypothalamic inflammatory markers and neuropeptides expression were also detected in all four groups. Results showed that LPS administration led to significant increase in hypothalamic inflammation together with muscle wasting. Increased hypothalamic neuropeptides, proopiomelanocortin (POMC), cocaine and amphetamine-related transcript (CART) and neuropeptides Y (NPY) and decreased agouti-related protein (AgRP) were also observed. Meanwhile dexmedetomidine administration ameliorated muscle wasting, hypothalamic inflammation and modulated the alteration of neuropeptides, POMC, CART and AgRP, in endotoxemic rats. In conclusion, dexmedetomidine could alleviate muscle wasting in endotoxemic rats, and it could also attenuate the alteration of hypothalamic neuropeptides and reduce hypothalamic inflammation.

## Introduction

Pain and anxiety are common in critically ill due to intubation, infection, trauma and long-term bedridden. When inappropriately treated, pain and anxiety can cause tachycardia, immunosuppression, increased oxygen consumption, elevated catecholamine production and metabolism[1]. So it is neccessary and beneficial to keep critically ill sedated[2]. Traditional sedative agents may generate unpredictable and prolonged duration of action in critically ill, due to the redistribution and accumulation of active metabolites[3]. Benzodiazepines have also been reported to be associated with increased risk of delirium, coma and respiratory depression[4]. Nowadays many guidelines recommend using non-benzodiazepines, like dexmedetomidine[5]. Dexmedetomidine, a new sedative, can cause analgesia and induce a sedative state similar to physiologic sleep without causing respiratory depression, by acting on α-2 receptors in the locus caeruleus[6]. Besides the benefits of shortening duration of mechanical ventilation and length of ICU stay, dexmedetomidine has also been demonstrated to reduce risk of delirium and hypertension[7,8]. Moreover in our previous study, we found that the addition of dexmedetomidine to analgesia for patients after abdominal operations could enhance recovery of gastrointestinal function, alleviate postoperative pain and lower metabolism[9]. However, the exact mechanism of these positive effecets on critically ill is still unkown.

Hypercatabolism exists in most critically ill patients, which contributes to serious complications and increased mortality[10]. Hypercatabolism can induce a major alteration in metabolism, including reduced appetite, increased catabolism and decreased anabolism of fat and proteins, which all lead to the prolonged phase of critical illness[11]. It is worth noting that unlike starvation or malnutrition, these metabolic changes could hardly be corrected by nutrition support alone. Although, much effort, for example glutamine supplement, growth factor treatment and anti-inflammatory lipid administration has been made to deal with the disordered metabolism, the efficacy is far from satisfactory[12,13]. As previously illustrated, protein catabolism and muscle wasting are thought to play an crucial role in the unsatisfactory efficacy of nutrition support and act as an important contributor to morbidity and mortality among all the metabolic alterations[14]. Thus it is urgent to find out the possible mechanism of hypercatabolism and explore new solutions for this dilemma.

Recently accumulative evidences suggested the importance of central nervous system (CNS) in the regulation of metabolism in disease states[15]. It is proved that central administration of several cytokines can recapitulate the response of peripheral LPS stimulation. In addition, Wisse et al. have demonstrated central inflammation was essential to elicit anorexia induced by peripheral LPS stimulation through bone marrow transplant methods[16]. It is also worth noting that dexmedetomidine generates sedative effects by acting on the locus caeruleus area, which is associated with stress response in CNS[1]. The locus caeruleus area is also the main area generating norepinephrine and can act on most areas of CNS, including hypothalamus. The hypothalamic arcuate nucleus(ARC) is considered to be an important site in the CNS to regulate metabolism and energy homeostasis[17], and the melanocortin system within the hypothalamus is recognized to be an important target of inflammation[18]. ARC is composed of two populations of neurons, POMC and AgRP neurons[19]. The former neuron expresses anorexigenic peptides, POMC and CART. POMC can be pyrolysed into α-melanocytestimulating hormone (α-MSH), which derives anorectic effects by binding to type-3 melanocortin receptor (MC3-R) and MC4-R. AgRP neuron expresses orexigenic peptides AgRP and NPY, which could stimulate appetite and decrease metabolism by acting as an inverse agonist of the MC4-R[20]. Both POMC and AgRP neurons are subject to pro-inflammatory cytokines. Studies have shown cytokines including interleukin (IL)-1β and IL-7 could increase POMC mRNA expression and decrease AgRP mRNA expression, which result in weight loss and muscle wasting[21]. Previously, our studies have suggested that hypothalamic peptides and inflammation may have participated in the endotoxemia-induced muscle wasting in rat models[22,23]. Based on this, we assume that apart from sedation and analgesia, dexmedetomidine could modulate metabolism and reduce muscle wasting through hypothalamic pathway. This may be another possible mechanism accounting for its positve effects on critically ill.

## Materials and methods

### Animals

Fourty-eight adult male Sprague–Dawley rats (250±20 g) were obtained from the Animal Research Center, Jinling Hospital, Nanjing, China. The animals were housed under regular lighting conditions (light cycle 600–1800) in a constant temperature environment(25°C) with free access to tap water and standard rat pellet chow. All animals used in this experiment were monitored two times a day, including bedding changes, water refill and food supplement.

Before the experiment, rats were given at least 7 days to adapt to the environment. The experimental protocols were approved by the Institutional Animal Care and Use Committee of Nanjing University and Jinling Hospital.

### Study protocol

Generally, all rats were anesthetized intraperitoneally with phenobarbital sodium (50 mg/kg) and then the femoral artery was cannulated to monitor study drugs. All rats were randomly categorized into four groups: CON (saline control), PF(pair fed), LPS-CON (endotoxemia + saline) and LPS-DEX (endotoxemia + dexmedetomidine), each group containing twelve animals. Animals in LPS-saline and LPS DEX groups were injected intraperitoneally with LPS (5 mg/kg, Escherichia coli serotype 055:B5, Sigma, St.Louis, MO, USA). Thirty minutes thereafter, LPS-DEX group received 50μg/kg dexmedetomidine(Hengrui Medicine Co.LTD, Jiangsu,China) administration via the femoral vein catheter (infusion at 5 μg·kg^-1^·hr^-1^) and LPS-CON group received equal amount of saline. The CON group were injected intraperitoneally with saline (5 mg/kg). The PF group was a subset of saline-treated rats pair fed to LPS injected rats to eliminate the effects of different food intake on central neuropeptides and inflammation. Due to the reproducibility, controllability and representativeness of LPS injection, it was widely used to study muscle wasting in endotoxemia; thus, we applied this method as previously described[24]. The dexmedetomidine used was short-acting intravenous medicine, mainly within 24 h. The dose of 50μg/kg of dexmedetomidine was chosen based on previous studies of the effects of dexmedetomidine in vivo[22]. After the administration of dexmedetomidine or saline, rats were then put back to the animal facility. Twenty-four hours later, the rats were executed with an overdose of phenobarbital sodium and the hypothalamus tissue was rapidly dissected and kept at -80°C until analysis. The extensor digitorum longus (EDL) was immediately obtained to measure the proteolytic rate, and the gastrocnemius muscle was harvested and frozen in -80°C.

### Rate of protein turnover

To measure protein breakdown rates, as formly described[23], fresh EDL muscles were fixed via the tendons to aluminium wire supports at resting length, and preincubated in oxygenated medium (95% O2–5% CO2): Krebs–Henseleit bicarbonate buffer (pH7-4) which contains 5 mM glucose, 0–1 U/ml insulin, 0–1 mM isoleucine, 0–17 mM leucine and 0–20 mM valine. After one hour preincubation, muscles were transferred to fresh medium of identical composition and incubated for a further 2 h with 0–5 mM cycloheximide. The degradation rates of total and myofibrillar proteins were measured by the release in the medium of free tyrosine and 3-methl-histidine (3-MH) respectively, and expressed as nanomoles of tyrosine/3-MH in medium per 2 h/g/muscle. Muscle was also homogenized in 0–4 mM perchloric acid to determine tissue-free 3-MH and tyrosine. The net generation of 3-MH was calculated as the amount of 3-MH in the medium minus the decrease in tissue free 3-MH before and after incubation. Net free tyrosine generation was calculated as the amount of tyrosine released into the medium plus the increase in tissue-free tyrosine during incubation. Both tyrosine and 3-MH levels in medium or tissue samples were measured by high-performance liquid chromatography (HPLC).

Double distilled water was used in HPLC. All reagent and water was filtered through a 0.45μm filter membrane. Derivating agent was prepared the same day it was used. After muscle tissues were thawed, take 200mg into homogeniser with 0.5mL absolute ethyl alcohol and homogenated at a high speed under 4°C for 10min. Followed by centrifugation at 12000rmp for 15min and reserve supernatant for HPLC. Precolumn derivatization, mix 10μl sample or reference standard with 100μl and reaction for 15min at room temperature. Then draw 20μl sample for computer analysis. Moblie phase was 50mmol/L sodium acetate solution, formaldehyde and tetrahydrofuran, of which the proportion of A liquid is 82:17:1 and B liquid is 22:77:1. Column temperature was at 30°C, flow velocity was 1.0mL/min. Excitation and emission wavelength of HPLC fluorescence detector was 338nm and 425nm respectively. Data was acquired and chromatograms were analyzed with Chromeleon 7.2 software.

### Analysis of cytokine gene expression in the muscle and hypothalamus tissues

The total RNA was isolated from hypothalamus and gastrocnemius muscle using Trizol reagent(Invitrogen, USA) according to the manufacturer’s instructions. The mRNAs of TNF-α and IL-6 were separately reverse-transcribed to cDNA and measured by real-time quantitative polymerase chain reaction (PCR), which was performed using a Rotor Gene 3000 system (Corbet, Sydney, Australia). Gene expression was analysed using the Rotor-Gene Real-Time Analysis Software 6.1. Glyceraldehyde phosphate dehydrogenase (GAPDH) was used as an internal control gene to normalize the target mRNAs, and gene expression was compared among groups using the DDCT method. The primer sequences are listed in Table 1.

**Table 1:** The primer sequences.

Gene		Primers	Accession No.
MuRF1	Forward	5’-GGACGGAAATGCTATGGAGA-3’	NM_080903.1
	Reverse	5’-AACGACCTCCAGACATGGAC-3’	
MAFbx	Forward	5’-CCATCAGGAGAAGTGGATCTATGTT-3’	NM_133521.1
	Reverse	5’-ATGACGTG AAACCCCCTTCG-3’	
POMC	Forward	5’-CCTCCTGCTTCAGACCTCCA-3’	NM_139326.2
	Reverse	5’-GGCTGTTCATCTCCGTTGC-3’	
AgRP	Forward	5’-TGAAGGGCATCAGAAGGT-3’	NM_033650.1
	Reverse	5’-CACAGGTCGCAGCAAGGT-3’	
CART	Forward	5’-CCGAGCCCTGGACATCTA-3’	NM_017110.1
	Reverse	5’-GGAATGCGTTTACTCTTGAGC-3’	
NPY	Forward	5’-CTGACCCTCGCTCTATCC-3’	NM_012614.2
	Reverse	5’-GGTCTTCAAGCCTTGTTCT-3’	
IL-1β	Forward	5’-TTCAAATCTCACAGCAGCAT-3’	NM_031512.2
	Reverse	5’-AGGTCGTCATCATCCCAC-3’	
TNF-α	Forward	5’-CCACGCTCTTCTGTCTACTG-3’	NM_012675.3
	Reverse	5’-GCTACGGGCTTGTCACTC-3’	
GAPDH	Forward	5’-GCAAGTTCAACGGCACAG-3’	NM_017008.4
	Reverse	5’-GCCAGTAGACTCCACGACAT-3’	

### Statistical analysis

The experimental data were expressed as means ± standard error (SE). Statistical analyses were performed using SPSS 21.0 software (SPSS Inc, USA). The comparisons of differences among groups were accomplished by a one-way analysis of variance(ANOVA), with pretreatment (LPS and saline) and treatment (dexmedetomidine and saline) as the main factors, followed by Newman–Keuls post hoc test. Differences were considered statistically significant at P < 0.05.

## Results

### Rate of protein breakdown and muscle atrophic gene expression

**Fig 1:**
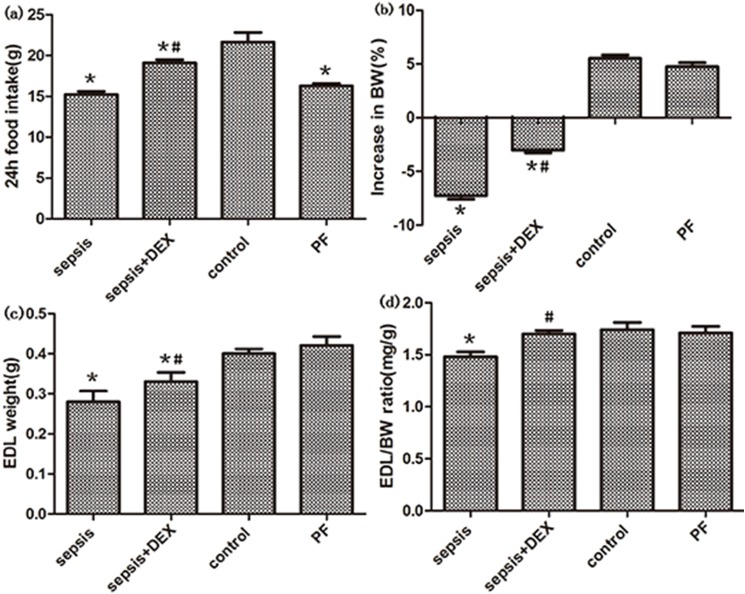
Effects of dexmedetomidine(DEX) and saline treatment on food intake, body weight (BW), muscle weight (MW) and MW:BW ratio in control and septic rats. (a,b), 24-h food intake and increased per cent of body weight were measured in four groups. (c) Weight of extensor digitorum longus (EDL) in four groups. (d) EDL weight was normalized to initial body weight (EDL:BW ratio) in four groups. n = 8 per group. Significant data measured from control were labelled (*) with P-values <0.05. Significant data measured from sepsis group was labelled (#) with P-values <0.05.

Compared with the control, LPS administration resulted in significant decreased food intake (P < 0.01, Fig 1), loss of body weight(BW) and lower EDL–BW ratio (both P < 0.05, Fig 1). In the PF group, no significant difference in body weight, EDL weight and EDL–BW ratio were observed when compared with the control, indicating that the decreased food intake had very little effect on muscle weight. However, in the group treated with LPS and dexmedetomidine, both weight loss and reduction of EDL-BW ratio were attenuated (P < 0.05 Fig 1). Original data can be seen in S1 Tables.

**Fig 2:**
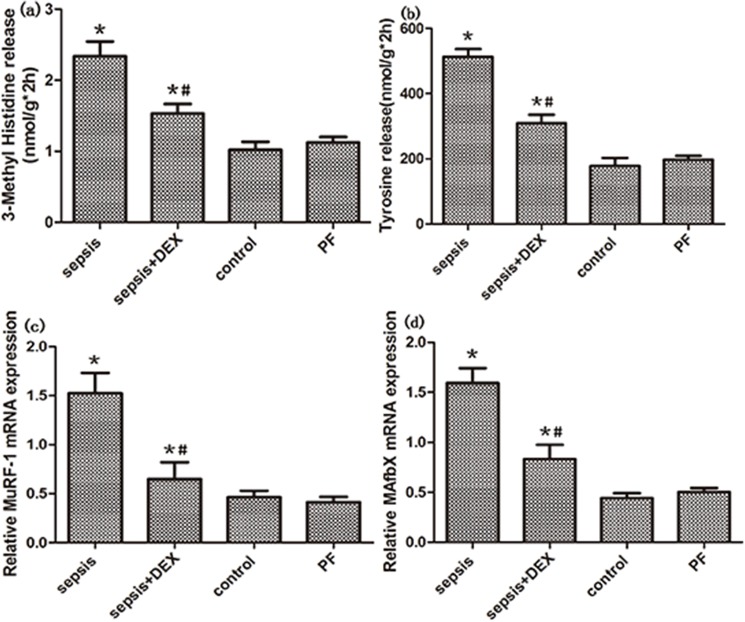
Total protein breakdown and muscle atrophic genes expression in control, septic and treated with dexmedetomidine(DEX) or saline rats. (a, b) 3-MH and tyrosine release were measure in four groups by HPLC. (c, d) The expression of MuRF-1 and MAFbx mRNA, measured by real-time PCR, was shown. GAPDH was used as control. n = 8 per group. Significant data measured from control was labelled (*) with P-values <0.05. Significant data measured from sepsis group were labelled (#) with P-values <0.05.

Muscle protein breakdown was measured by 3-MH and tyrosine release. As expected, when compared with the control, there was a significant increase in the rate of total protein proteolysis 24 hours after LPS administration(both P < 0.01, Fig 2). Also, significant elevated expression of two atrophic gene, MuRF-1 and MAFbx was obsevred after LPS administration (P < 0.01, Fig 2). These results confirmed that endotoxemia could induce muscle wasting in animal models. Despite decreased food intake has little effect on muscle wasting in saline pretreated rats as shown in PF group, dexmedetomidine administration could significantly attenuate total protein breakdown in endotoxemic rats (P < 0.01, Fig 2). Similarly, the mRNA expression of MuRF-1 and MAFbx in endotoxemic rats were significantly reduced when treated with dexmedetomidine(both P < 0.05, Fig 2). Original data can be seen in S1 Tables. Taken together, these results demonstrated that dexmedetomidine treatment can alleviated the muscle wasting caused by endotoxemia.

### Hypothalamic neuropeptides and inflammatory cytokine gene expression

**Fig 3:**
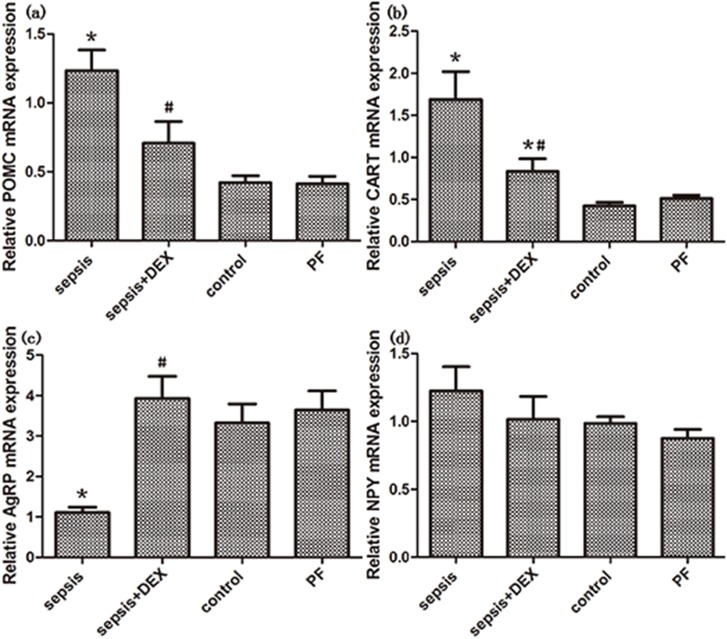
Effects of dexmedetomidine(DEX) and saline treatment on the expression of hypothalamic neuropeptides, POMC, CART, AgRP and NPY, in control, septic and treated (with DEX or saline) rats. Hypothalamic genes expression were measured by real-time PCR. GAPDH was used as control. All the mRNA expressions were normalized to those of control group. Significant data measured from control were labelled (*) with P-values <0.05. Significant data measured from sepsis group was labelled (#) with P-values <0.05.

The anorexigenic genes, POMC and CART, expression increased after LPS administration when compared with the control and PF group(both P < 0.05, Fig 3). On the contrary, there was a significant decrease in orexigenic neuropeptide AgRP expression in endotoxemic rats(P<0.01, Fig 3). However, the expression of another orexigenic neuropeptide, NPY, did not decrease after LPS administration (Fig 3). In the group treated with LPS and dexmedetomidine, downregulated expression of POMC and CART was observed when compared with endotoxemia group (both P < 0.05, Fig 3), although they did not reach the level of control group. Additionaly, the reduction of AgRP caused by LPS was reversed (P < 0.01, Fig 3) by dexmedetomidine and showed no significant difference comparing with the control group (P > 0.05, Fig 3). However, dexmedetomidine administration showed little effect on the increased expression of NPY induced by LPS administration(P>0.05, Fig 3). Original data can be seen in S1 Tables.

**Fig 4:**
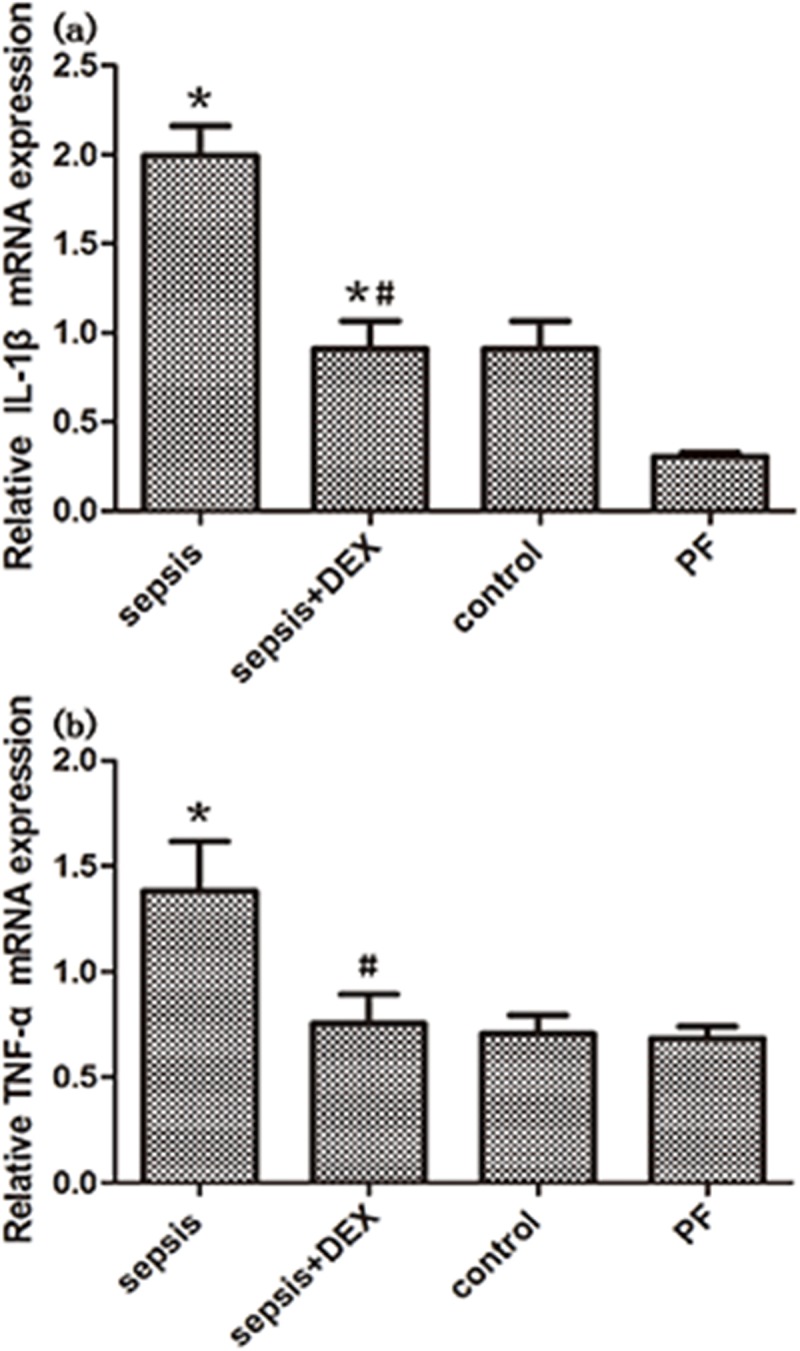
Expression of hypothalamic inflammatory genes, IL-1band TNF-a in control, septic and treated with dexmedetomidine(DEX) or saline rats. Inflammatory genes expression was measured by real-time PCR. GAPDH was used as control. All the mRNA expressions were normalized to those of control group. n = 8 per group. Significant data measured from control was labelled (*) with P-values <0.05. Significant data measured from sepsis group were labelled (#) with P-values <0.05.

Significant elevation of hypothalamic inflammatory markers, IL-1β and TNF-α, were observed after LPS administration when compared with the control(P < 0.01, Fig 4). And dexmedetomidine administration could attenuated the increased hypothalamic inflammation induced by LPS, it is worth noting that the level of TNF-α almost returned to normal(P>0.05, Fig 4). Original data can be seen in S1 Tables. These results indicated that dexmedetomidine might have a hypothalamic anti-inflammatory effect in endotoxemic rats.

## Discussion

It is well konwn that many factors could influence metabolism. For example, metabolism would elevate under the circumstances of pain or anxious. Due to intubation, infenction, trauma and long-term bedridden, pain and anxiety exist in most critically ill patients. When inappropriately treated, pain and anxiety can cause many negative effects, so it is neccessary and beneficial to keep critically ill patients sedated[2]. Compared with tradional benzodiazepine sedatives, non-benzodiazepines, like midazolam and dexmedetomidine, may be more applicable and safer[5]. Dexmedetomidine is a highly selective α-2 adrenoreceptor agonist providing sedative and anxiolytic activity via receptors within the locus ceruleus, analgesia via receptors in the spinal cord, and attenuation of the stress response with no significant respiratory depression[6]. It proved to be safer by causing fewer adverse effects and could reduce cardiac output and hepatic blood flow, potentially increasing its action duration in critically ill patients[25]. Although dexmedetomidine was generally used for sedation in critically ill patients, our previous clinical study found that applying dexmedetomidine to postoperative patients could accelerate the recovery of gastrointestinal function and lower metabolism[11]. However, the exact mechanism of dexmedetomidine’s positive effecets on critically ill patients remains unkown. Here we first demonstrated that dexmedetomidine treatment could reduce endoxemia-induced muscle wasting and is associated with the alteration of hypothalamic peptides and inflammation.

As shown in the present study, dexmedetomidine administration significantly reduced the release of 3-MH and tyrosine and decreased the expression of two important muscle atrophy gene in endotoxemic rats, MuRF-1 and MAFbx. In other words, endotoxemic muscle wasting was attenuated. Richard and others demonstrated that dexmedetomidine-treated patients spent less time on mechanical ventilation[26]. Prolonged mechanical ventilation in critically ill patients was considered to be associated with the atropy of resparitory muscles, including intercostal muscles, diaphragm, and the abdominal muscles. With combination of our results, dexmedetomidine alleviating endoxemia-induced muscle wasting could be one of the important mechanism for its ability to shorten duration of mechanical ventilation in critically ill.

However, the underlying mechanism of dexmedetomidine’s ability to reduce endotoxemic muscle wasting was undefined so far. Dexmedetomidine generates sedative effects by acting on the locus caeruleus area, which is associated with stress response in the brain[1]. And the locus caeruleus area is the main area generating norepinephrine and can act on most sites in the CNS, including hypothalamus. So we assume that dexmedetomidine may have the effect of metabolic modulation through hypothalamic pathway and here we first determined the hypothalamic effect of dexmedetomidine in endotoxemic models. In the present study, we found that in endotoxemic rats, dexmedetomidine could attenuate the alterations of hypothalamic peptides, accompanied by lower skeletal muscle wasting. These results indicated dexmedetomidine attenuating muscle wasting might be associated with the regulation of hypothalamic neuropeptides. The exact mechanism of how dexmedetomidine regulated neuropeptides in endotoxemia still needed further investigation.

Pro-inflammatory cytokines, endothelium-released oxygen radicals and complement factors contributed to the systemic inflammation in endotoxemic or septic states. In the past, peripheral inflammation was considered as a major factor accounting for muscle wasting or even organ dysfunction[27]. However, clinical trials with administration of TNF-α antibody or steroids showed negative results in septic patients[28,29], indicating that peripheral inflammation may not be as important as we used to think in the process. Meanwhile, there are emerging studies suggested that inflammation in the CNS may play a more important role in the endotoxemic or septic muscle wasting and negative energy balance than peripheral inflammation[30,31]. In our study, LPS administration resulted in increased hypothalamic inflammatory cytokines, which was in consistent with previous studies.

The constant hypothalamic inflammation could have a negative impact on the energetic balance mainly through the melanocortin system. A series of studies have shown that the melanocortin system was invloved in the regulation of malignant consumption or even cachexia. While the cachexia syndrome could be ameliorated by blocking MC4-R signalling using genetic or pharmacological methods in renal failure, cardiac disease and acute LPS injection[32]. Moreover, reduced muscle wasting was observed after administration of AgRP, the antagonist of MC4-R, in the animal model of chronic kidney disease (CKD)[33]. Thus on the other hand, the decreased endotoxemia-induced muscle wasting observed in the present study could be the result of neuropeptides alteration induced by central inflammation.

In the endotoxemia group in our study, the hypothalamic inflammation was also related to the alterations of the mRNA expression of POMC and AgRP, which was consistent with former studies[25]. It has been proved that POMC and AgRP neurones both expressed IL-1 receptors, making the two neurons subject to inflammatory cytokines[30]. Thus stimulated by IL-1β, which was be activated in endotoxemic or septic central inflammation, increased POMC and decreased AgRP expression were observed. This was further supported by the present results.

As a matter of fact, in addition to sedation, dexmedetomidine can reduce systemic inflammation significantly[25], which could also account for the benefits observed after dexmedetomidine administration in critically ill with sepsis. In the present study, we found that hypothalamic inflammation was attenuated after dexmedetomidine administration, which may contribute to the neuropeptides alteration. Apart from acting on the locus caeruleus area[1], the transportation of dexmedetomidine from blood circulation to CNS was elevated in septic models due to increased permeability of blood brain barrier. Thus, the anti-inflammation effect of dexmedetomidine was very likely to be achieved in the CNS. Hypothalamic inflammation could lead to muscle degradation and atrophy[31], mainly through the melanocortin system, so the regulative effect of dexmedetomidine on hypothalamic neuropeptides may also contribute to the alleviated muscle wasting in endotoxemia and its ability to shorten duration of mechanical ventilation in critically ill.

Hypercatabolism exists in most critically ill patients with severe trauma, burn or sepsis, which contributes to the prolonged mechanical ventilation, infection and ICU stay in critically ill. Muscle wasting was considered to be the main characteristic of hypercatabolism and could result in severe complications and increase mortality[33]. Many interventions, including early enteral nutrition and trophic enteral feeding[34,35] have been proposed to deal with this dilemma, the effect was still unsatisfactory. Recently, Puthucheary et al. have demonstrated that muscle wasting in critically ill raised on admission and remained high during the first week of ICU stay[20]. More importantly, the alteration in timing and type of nutrition had little or no effect and, even could aggravated the acute muscle loss in some cases[11]. Mechanism studies have shown that increased protein degradation other than decreased protein synthesis, counted for muscle wasting[20].

Here we found that dexmedetomidine could alleviate endotoxemia-induced muscle wasting and affect hypothalamic peptides simultaneously, which could be interpreted as an important complementary mechanism of dexmedetomidine’s positive effects on critically ill. Moreover, this could provide a new perspective to solve the problem of hypercatabolism and improve the effect of nutritioanl support in critically ill patients. In the future, we should pay more attention to the CNS conditions to evaluate metabolism in septic patients in clinical practise. Or perhaps combining with central-oriented pharmaceuticals, dexmedetomidine administration may achieve better results. But all of these presumptions need further investigations to validate.

Although here we demonstrated that dexmedetomidine may attenuate muscle wasting through a central circuit, there were some limitations. Firstly, we only utilized peripheral dexmedetomidine administration to investigate its central action. Despite the resemblance of clinical practice, it was compromised when compared with central administration. Secondly, as a sedative drug, dexmedetomidine can reduce activity, alleviate pain and anxiety, which could all lead to lower metabolism directly. So the role that hypothalamic neuropeptides played may not completely account for the reduced muscle wasting we observed. Thirdly, we only investigated the effect of dexmedetomidine on hypothalamus and muscles at 24 h after LPS injection. Longer time effect is required in the future studies. Also, detailed molecular pathway is needed to show the exact mechanism of dexmedetomidine’s effect on central melanocortin system under septic conditions.

## Conclusions

In conclusion, dexmedetomidine could reversing muscle wasting, and it could also attenuate the alteration of hypothalamic neuropeptides and reduce hypothalamic inflammation. This expanded the usage of dexmedetomidine in critically ill besides sedation. The exact mechanism for dexmedetomidine’s central effect on endotoxemia-induced muscle wasting requires further exploration. These results may provide a new perspective for the research and management of muscle wasting in critically ill patients and the study of dexmedetomidine effect.

## Supporting information

S1 Tables (DOCX)
