# Supplementary material for: Dexmedetomidine ameliorates muscle wasting and attenuates the alteration of hypothalamic neuropeptides and inflammation in endotoxemic rats
Source: PLoS One. 2017 Mar 30;12(3):e0174894. doi: 10.1371/journal.pone.0174894 (PMC5373637; doi:10.1371/journal.pone.0174894)
Supplement: S1 Tables — (DOCX) [file pone.0174894.s001.docx]

**Table 1. 24h food intake(g)**

|  | sepsis | Sepsis+DEX | control | PF |
| --- | --- | --- | --- | --- |
| 1 | 16.32 | 18.84 | 23.43 | 17.34 |
| 2 | 16.89 | 19.54 | 20.58 | 16.82 |
| 3 | 14.35 | 20.53 | 19.42 | 15.78 |
| 4 | 13.57 | 20.41 | 25.65 | 13.69 |
| 5 | 15.63 | 17.94 | 28.25 | 16.56 |
| 6 | 14.98 | 18.47 | 21.86 | 17.29 |
| 7 | 15.73 | 17.34 | 19.98 | 15.83 |
| 8 | 14.37 | 19.65 | 17.29 | 15.30 |
| 9 | 15.64 | 16.43 | 20.12 | 16.75 |
| 10 | 17.02 | 18.90 | 22.34 | 16.23 |
| 11 | 14.72 | 21.55 | 18.49 | 18.45 |
| 12 | 13.52 | 19.48 | 22.39 | 15.32 |

**Table 2. Increase in BW(%)**

|  | sepsis | Sepsis+DEX | control | PF |
| --- | --- | --- | --- | --- |
| 1 | -8.21 | -3.45 | 5.64 | 5.02 |
| 2 | -7.12 | -2.87 | 6.03 | 4.87 |
| 3 | -6.57 | -3.59 | 4.96 | 3.32 |
| 4 | -7.36 | -1.99 | 4.32 | 4.05 |
| 5 | -8.01 | -2.56 | 5.98 | 4.78 |
| 6 | -5.62 | -4.05 | 4.83 | 5.43 |
| 7 | -6.89 | -3.28 | 5.32 | 3.98 |
| 8 | -8.30 | -2.45 | 7.16 | 6.63 |
| 9 | -7.19 | -2.35 | 5.98 | 5.64 |
| 10 | -6.58 | -3.14 | 6.12 | 3.96 |
| 11 | -9.02 | -2.65 | 5.23 | 4.69 |
| 12 | -6.25 | -3.98 | 4.79 | 4.75 |

**Table 3. EDL weight(g)**

|  | sepsis | Sepsis+DEX | control | PF |
| --- | --- | --- | --- | --- |
| 1 | 0.34 | 0.34 | 0.41 | 0.39 |
| 2 | 0.22 | 0.24 | 0.37 | 0.49 |
| 3 | 0.19 | 0.39 | 0.38 | 0.43 |
| 4 | 0.32 | 0.31 | 0.27 | 0.39 |
| 5 | 0.21 | 0.38 | 0.46 | 0.34 |
| 6 | 0.30 | 0.28 | 0.36 | 0.44 |
| 7 | 0.25 | 0.27 | 0.39 | 0.37 |
| 8 | 0.41 | 0.43 | 0.40 | 0.52 |
| 9 | 0.19 | 0.29 | 0.52 | 0.46 |
| 10 | 0.34 | 0.35 | 0.45 | 0.38 |
| 11 | 0.23 | 0.32 | 0.36 | 0.51 |
| 12 | 0.36 | 0.36 | 0.43 | 0.32 |

**Table 4. EDL/BW(mg/g)**

|  | sepsis | Sepsis+DEX | control | PF |
| --- | --- | --- | --- | --- |
| 1 | 1.67 | 1.68 | 1.77 | 1.73 |
| 2 | 1.50 | 1.72 | 1.85 | 1.62 |
| 3 | 1.43 | 1.59 | 1.58 | 1.58 |
| 4 | 1.32 | 1.82 | 1.52 | 1.82 |
| 5 | 1.56 | 1.73 | 2.09 | 1.64 |
| 6 | 1.49 | 1.81 | 1.84 | 1.53 |
| 7 | 1.60 | 1.54 | 1.49 | 1.67 |
| 8 | 1.27 | 1.71 | 1.78 | 2.09 |
| 9 | 1.34 | 1.64 | 1.79 | 1.87 |
| 10 | 1.43 | 1.82 | 1.53 | 1.58 |
| 11 | 1.61 | 1.57 | 1.88 | 1.66 |
| 12 | 1.54 | 1.77 | 1.76 | 1.73 |

**Table 5.** **3-MH release(nmol/g*2h)**

|  | sepsis | Sepsis+DEX | control | PF |
| --- | --- | --- | --- | --- |
| 1 | 1.979 | 1.769 | 1.421 | 1.212 |
| 2 | 3.026 | 1.880 | 0.883 | 0.983 |
| 3 | 2.508 | 1.396 | 0.912 | 1.341 |
| 4 | 1.257 | 1.735 | 1.131 | 0.761 |
| 5 | 2.543 | 0.967 | 1.523 | 1.21 |
| 6 | 1.981 | 0.992 | 0.756 | 0.892 |
| 7 | 2.453 | 1.570 | 0.912 | 1.239 |
| 8 | 2.974 | 1.939 | 0.641 | 1.354 |
| 9 | 2.432 | 1.674 | 0.991 | 1.268 |
| 10 | 1.876 | 1.876 | 1.324 | 1.042 |
| 11 | 2.229 | 0.983 | 1.132 | 0.992 |
| 12 | 2.798 | 1.591 | 0.71 | 1.194 |

**Table 6.** **Tyrosine release(nmol/g*2h)**

|  | sepsis | Sepsis+DEX | control | PF |
| --- | --- | --- | --- | --- |
| 1 | 609.12 | 431.55 | 200.25 | 211.5 |
| 2 | 498.23 | 289.64 | 204.35 | 174.77 |
| 3 | 503.12 | 372.54 | 185.36 | 163.78 |
| 4 | 412.74 | 287.43 | 104.56 | 238.54 |
| 5 | 603.21 | 312.34 | 148.43 | 152.18 |
| 6 | 523.85 | 197.99 | 115.98 | 199.49 |
| 7 | 498.2 | 343.51 | 143.55 | 241.09 |
| 8 | 457.45 | 238.28 | 323.36 | 198.25 |
| 9 | 612.32 | 298.43 | 212.57 | 202.43 |
| 10 | 478.37 | 372.18 | 193.75 | 136.79 |
| 11 | 542.25 | 335.76 | 143.23 | 239.42 |
| 12 | 420.02 | 230.27 | 163.37 | 211.16 |

**Table 7. Relative MuRF-1 mRNA expression**

|  | sepsis | Sepsis+DEX | control | PF |
| --- | --- | --- | --- | --- |
| 1 | 1.85 | 0.164 | 0.674 | 0.338 |
| 2 | 2.452 | 0.378 | 0.138 | 0.352 |
| 3 | 0.816 | 0.269 | 0.453 | 0.631 |
| 4 | 1.854 | 0.876 | 0.379 | 0.286 |
| 5 | 0.978 | 1.236 | 0.502 | 0.482 |
| 6 | 1.476 | 0.422 | 0.365 | 0.641 |
| 7 | 2.343 | 0.377 | 0.476 | 0.356 |
| 8 | 1.891 | 1.470 | 0.725 | 0.22 |
| 9 | 1.763 | 1.115 | 0.518 | 0.554 |
| 10 | 0.942 | 0.537 | 0.276 | 0.377 |
| 11 | 1.048 | 0.629 | 0.395 | 0.283 |
| 12 | 0.875 | 0.315 | 0.667 | 0.352 |

**Table 8. Relative MAfbX mRNA expression**

|  | sepsis | Sepsis+DEX | control | PF |
| --- | --- | --- | --- | --- |
| 1 | 2.152 | 0.243 | 0.534 | 0.554 |
| 2 | 1.896 | 0.588 | 0.386 | 0.371 |
| 3 | 0.866 | 1.364 | 0.287 | 0.612 |
| 4 | 1.325 | 1.025 | 0.604 | 0.486 |
| 5 | 1.382 | 0.424 | 0.465 | 0.399 |
| 6 | 1.439 | 1.321 | 0.413 | 0.683 |
| 7 | 1.732 | 0.864 | 0.621 | 0.514 |
| 8 | 2.707 | 0.843 | 0.234 | 0.405 |
| 9 | 0.959 | 1.102 | 0.387 | 0.563 |
| 10 | 1.038 | 0.915 | 0.594 | 0.679 |
| 11 | 1.672 | 0.358 | 0.289 | 0.381 |
| 12 | 1.960 | 0.961 | 0.502 | 0.389 |

**Table 9. Relative POMC mRNA expression**

|  | sepsis | Sepsis+DEX | control | PF |
| --- | --- | --- | --- | --- |
| 1 | 1.001 | 0.346 | 0.653 | 0.385 |
| 2 | 2.055 | 0.735 | 0.365 | 0.432 |
| 3 | 1.255 | 1.235 | 0.298 | 0.613 |
| 4 | 0.603 | 0.363 | 0.438 | 0.329 |
| 5 | 1.256 | 0.256 | 0.512 | 0.297 |
| 6 | 1.523 | 0.578 | 0.502 | 0.631 |
| 7 | 0.986 | 0.634 | 0.398 | 0.443 |
| 8 | 1.193 | 1.509 | 0.202 | 0.166 |
| 9 | 1.092 | 1.007 | 0.389 | 0.289 |
| 10 | 1.993 | 0.744 | 0.512 | 0.477 |
| 11 | 0.822 | 0.613 | 0.279 | 0.610 |
| 12 | 1.029 | 0.464 | 0.504 | 0.272 |

**Table 10. Relative AgRP mRNA expression**

|  | sepsis | Sepsis+DEX | control | PF |
| --- | --- | --- | --- | --- |
| 1 | 0.634 | 1.901 | 2.756 | 5.093 |
| 2 | 1.107 | 5.135 | 3.451 | 4.231 |
| 3 | 1.893 | 2.895 | 4.253 | 2.674 |
| 4 | 1.113 | 1.952 | 2.186 | 3.023 |
| 5 | 0.864 | 6.523 | 5.031 | 2.601 |
| 6 | 0.932 | 3.873 | 1.824 | 1.954 |
| 7 | 1.231 | 6.012 | 1.982 | 5.823 |
| 8 | 1.148 | 5.175 | 5.101 | 3.761 |
| 9 | 0.982 | 4.287 | 4.284 | 4.231 |
| 10 | 0.876 | 1.899 | 2.223 | 2.874 |
| 11 | 1.453 | 3.023 | 1.991 | 4.927 |
| 12 | 1.555 | 4.521 | 3.702 | 2.548 |

**Table 11. Relative CART mRNA expression**

|  | sepsis | Sepsis+DEX | control | PF |
| --- | --- | --- | --- | --- |
| 1 | 3.329 | 0.336 | 0.601 | 0.632 |
| 2 | 1.995 | 0.491 | 0.365 | 0.556 |
| 3 | 0.99 | 1.078 | 0.442 | 0.341 |
| 4 | 1.153 | 1.707 | 0.508 | 0.387 |
| 5 | 1.001 | 0.823 | 0.531 | 0.497 |
| 6 | 1.213 | 0.61 | 0.275 | 0.461 |
| 7 | 0.972 | 0.753 | 0.384 | 0.552 |
| 8 | 2.858 | 0.882 | 0.278 | 0.662 |
| 9 | 1.783 | 0.981 | 0.299 | 0.701 |
| 10 | 0.893 | 0.678 | 0.387 | 0.528 |
| 11 | 2.021 | 1.109 | 0.671 | 0.396 |
| 12 | 2.12 | 0.572 | 0.335 | 0.419 |

**Table 12.** **Relative NPY mRNA expression**

|  | sepsis | Sepsis+DEX | control | PF |
| --- | --- | --- | --- | --- |
| 1 | 2.424 | 0.52 | 1.043 | 0.923 |
| 2 | 1.149 | 0.664 | 1.212 | 0.715 |
| 3 | 1.148 | 1.422 | 0.976 | 0.674 |
| 4 | 1.137 | 1.907 | 0.783 | 1.034 |
| 5 | 0.974 | 0.614 | 0.907 | 0.745 |
| 6 | 0.876 | 1.244 | 0.96 | 0.827 |
| 7 | 1.239 | 0.834 | 0.892 | 1.231 |
| 8 | 0.837 | 0.915 | 0.883 | 0.851 |
| 9 | 0.912 | 0.782 | 0.784 | 1.115 |
| 10 | 1.124 | 1.513 | 1.322 | 0.914 |
| 11 | 1.942 | 0.983 | 0.923 | 0.692 |
| 12 | 0.914 | 0.782 | 1.123 | 0.779 |

**Table 13. Relative IL-1β mRNA expression**

|  | sepsis | Sepsis+DEX | control | PF |
| --- | --- | --- | --- | --- |
| 1 | 2.134 | 0.277 | 0.077 | 0.301 |
| 2 | 1.375 | 0.635 | 0.035 | 0.331 |
| 3 | 1.91 | 1.043 | 0.043 | 0.198 |
| 4 | 2.456 | 1.342 | 0.142 | 0.287 |
| 5 | 1.642 | 0.498 | 0.698 | 0.22 |
| 6 | 1.623 | 1.276 | 0.076 | 0.401 |
| 7 | 2.017 | 1.478 | 0.378 | 0.367 |
| 8 | 2.803 | 0.731 | 0.131 | 0.343 |
| 9 | 1.673 | 0.873 | 0.092 | 0.411 |
| 10 | 2.157 | 1.231 | 0.512 | 0.209 |
| 11 | 1.844 | 0.762 | 0.173 | 0.322 |
| 12 | 2.306 | 0.774 | 0.415 | 0.282 |

**Table 14. Relative TNF-α mRNA expression**

|  | sepsis | Sepsis+DEX | control | PF |
| --- | --- | --- | --- | --- |
| 1 | 2.849 | 0.54 | 0.923 | 0.543 |
| 2 | 1.155 | 0.383 | 0.656 | 0.753 |
| 3 | 0.709 | 1.108 | 0.78 | 0.89 |
| 4 | 1.202 | 1.013 | 1.002 | 0.651 |
| 5 | 1.453 | 1.32 | 0.432 | 0.523 |
| 6 | 1.012 | 0.746 | 0.778 | 0.498 |
| 7 | 0.993 | 0.783 | 0.803 | 0.663 |
| 8 | 1.691 | 0.147 | 0.258 | 0.935 |
| 9 | 1.276 | 0.782 | 0.623 | 0.709 |
| 10 | 0.895 | 1.211 | 0.861 | 0.417 |
| 11 | 1.423 | 0.531 | 0.477 | 0.705 |
| 12 | 1.938 | 0.496 | 0.855 | 0.897 |
